# Chronic pain in primary care. German figures from 1991 and 2006

**DOI:** 10.1186/1471-2458-9-299

**Published:** 2009-08-18

**Authors:** Christine H Frießem, Anne Willweber-Strumpf, Michael W Zenz

**Affiliations:** 1 Department of Anaesthesiology, Intensive Care, Palliative Care and Pain Medicine BG-Universitätsklinikum Bergmannsheil – University Hospital, Ruhr-University, Bochum, Germany; 2 Pain clinic, Department of Anaesthesiology, Emergency and Intensive Care Medicine, Medical Centre University of Goettingen, Germany; 3 Department of Anaesthesiology, Intensive Care and Pain Medicine Knappschaftskrankenhaus Langendreer – University Hospital, Ruhr-University, Bochum, Germany

## Abstract

**Background:**

Until now only limited research has been done on the prevalence of chronic pain in primary care. The aim of this investigation was to study the health care utilisation of patients suffering from pain. How many patients visit an outpatient clinic because of the symptom of pain? These data were compared with data from a similar study in 1991, to investigate whether improvements had been achieved.

**Methods:**

A total of 1201 consecutive patients visiting outpatient clinics were surveyed in six practices in the western part of Germany on randomly selected days by means of questionnaires. Topics were the point prevalence of pain and the period prevalence of chronic pain, its characteristics and its impact on daily life, as well as data on previous therapies for pain. A retrospective comparison was made with the data from a similar study with same design surveying 900 patients that took place in five practices during 1991.

**Results:**

In 2006, pain was the main reason for consulting a doctor in 42.5% of all patients (1991: 50.3%). Of all respondents, 62% suffered from pain on the particular day of the consultation, and 40% reported that they had been suffering from pain for more than six months (1991: 36.4%). As many as 88.3% of patients with chronic pain reported a negative impact on their daily life due to this pain (1991: 68%), and 88.1% reported impairment of their working life because of chronic pain (1991: 59.1%).

**Conclusion:**

Pain, and chronic pain in particular, is a central problem in primary care. Over the last 15 years, the number of patients suffering from chronic pain has not decreased. In nearly half of all cases, pain is still the reason for health care utilisation in outpatient clinics. Pain represents a major primary health care problem with enormous impact on public health. Improvements can only be achieved by improving the quality of health care at the primary care level.

## Background

It is estimated that at least 8 to 10 million patients in Germany (around 10% of the population) suffer from chronic pain. Approximately 500 000 to 800 000 patients have difficult and complex pain problems demanding specialist treatment [1].

In the last few years, several epidemiological studies have been carried out. It is difficult to draw any reliable conclusions on the course and development of chronic pain in the outpatient care system. The direct comparison of study results over the years is hampered by heterogeneous populations, and by differences in sample sizes, randomisation techniques, methods, definitions of chronic pain and different estimates for prevalence levels. Consequently, data have revealed significant method-specific differences in the prevalence of chronic pain.

Epidemiological population-based studies report a pain prevalence of between 2% and 46.5% depending on the investigation and definition [2-6]. Interviews in the practices of both general practitioners (GPs) and specialists have indicated a variable and higher prevalence of between 22% and 50.4% [3,7-9]. Pain has become the most prevalent symptom in patients seeking medical advice, and is one of the main issues in public health [10,11].

Fifteen years after the first investigation [9], a similar study was designed and carried out in primary care in the same area of Germany. So the two data sets are really comparable and give hints as to changes. The aim of this study was to demonstrate the actual primary care utilisation of patients suffering from pain as the leading symptom. Additionally, the investigation aimed to look at trends over time and to demonstrate possible improvements in the outpatient care of pain patients resulting from the effort and actions of national and international pain societies.

## Methods

### Collection of epidemiological data

At the beginning of 2006, six different practices in Bochum were selected as representative of the different disciplines for the diagnosis and treatment of pain (general medicine with a focus on homoeopathy, surgery, internal medicine, neurology, oncology and orthopaedics). Upon approval of the ethics committee of the Medical Faculty of the Ruhr University Bochum, on randomly selected days all consecutive patients of all age groups entering these practices were asked to participate. Exclusion criteria were poor health, concerns about privacy and inability to understand the German questionnaire. It was planned to include up to 200 patients from each practice.

General information about the survey was provided by a flyer distributed at the reception desk. In the waiting room, all patients were informed about the aim and purpose of the survey and how to fill in the questionnaire. Once they had given their consent, patients were asked to complete the self-administered questionnaire. In cases of difficulty in understanding a question, assistance was provided by the same person, who had a medical background. All questionnaires were anonymous.

An investigation featuring the same study design had already been carried out in 1991 [9,12], and the same practices were used in the second investigation. In addition, the practice of a specialist in oncology was added in 2006, to include the aspect of cancer pain. In order to ensure comparability of the data of both investigations, the 2006 questionnaire was identical to the previous one [12]. By this means, prospective data from 2006 and retrospective data from 1991 could be compared.

### Structure of questionnaire

The questionnaire consisted of two different parts. [Additional file 1]. Part A collected demographic and social data and was answered by all patients who agreed to participate. Point prevalence of pain was revealed with the question: Do you suffer from pain today? Part B was only answered by those patients who suffered from chronic pain – irrespective of whether pain was the reason for the current visit to the doctor representing a period prevalence. Detailed data (Part B) were obtained if pain was the primary or secondary reason for the visit to the doctor. Chronic pain was defined as recurring or constant pain lasting longer than six months [13-17]. Acute pain was defined as pain lasting for hours or days.

The denominators of the data collected in Part B included only patients who reported chronic pain. This part of the questionnaire contained the characteristics, duration and localisation of the pain. The intensity of pain was reported by means of an eleven-point numerical rating scale (NRS) ranging from 0 to 10 (0 = no pain, 10 = worst possible pain). Using multiple-choice questions, patients were asked about the impact of pain on their daily activities, sleep and respective employment status. Other questions dealt with previous therapies and their subjective effects. The questionnaire included two open questions about profession and diagnosis as well as multiple-choice questions. Multiple answers were allowed for certain questions (for example, pain location, previous therapies and their subjective effects, and the daily impact of chronic pain).

### Statistical evaluation

Statistical differences between the results of 1991 and 2006 were calculated by means of the chi-square cross tabulation test, using the statistical software StatView Version 5.0 for Macintosh and Windows. Statistical significance was considered at p < 0.05.

## Results

In this section all results from 2006 are presented. For clear discrimination the figures from 1991 together with the significances are given in brackets following.

### Demographic aspects

In total 1201 questionnaires were completed (N = 1201) except for a limited number of questions (1991: n = 900). The refusal rate was 4.61%. The reason for exclusion was not always given. Only two patients were excluded because of poor health conditions. Both were not suffering from acute or chronic pain. Most of the excluded cases were related to the Turkish societies having difficulties with the German questionnaire. Women answered 61.7% of the questionnaires (1991: 60%). The mean age of all respondents was 53.4 years (1991: 48.6 years). The mean age of patients suffering from pain was 58.4 years. [Table 1].

**Table 1:** Basic data: Comparison of the surveys of 1991 and 2006

	1991	2006
**Number of practices **(n)	5	6

**Number of patients **(N)	900	1201

**women **(n/%)	540/59.9	741/61.7

**men **(n/%)	360/40.1	460/38.3

**mean age total **(in years)	48.6	53.4

**Patients with acute pain **(n/%)	228/25.3	265/22.1

**Patients with chronic pain **(n/%)	328/36.4	480/40.0

**women **(n/%)	219/66.8	324/67.5

**men **(n/%)	109/33.2	156/32.5

**mean age total **(in years)	-	58.4

**Actual consultation due to pain **(n/%)	453/50.3**	510/42,5**

**Actual consultation due to acute pain **(n/%)	228/25.3**	227/18.9**

**Actual consultation due to chronic pain **(n/%)	225/25.0	283/23.6

- missing data; ** p < 0.001

### Social data

More than half of the patients (55.6%) suffering from chronic pain were married (1991: 57%) and 9.4% of respondents with chronic pain were divorced (1991: 6.7%). Of the chronic pain sufferers, 43.6% had a general certificate of secondary education (grade 9 at any secondary school; 1991: 56.3%; p = 0.0005) while 23.5% of them had Abitur (corresponding to A-levels; 1991: 17.8%). The open question about profession could not be analysed because of missing answers. It was excluded to guarantee full comparability between the two questionnaires.

### Prevalence of pain

Pain was the main reason for the visit to the outpatient clinic in 42.5% of all participating patients in 2006 (1991: 50.3%; p = 0.0003). A total of 62% of respondents suffered from acute or chronic pain on the day of the survey 2006 (1991: 61.7%). 40% (n_2006 _= 480) suffered from chronic pain (1991: 36.4%; n_1991 _= 328). The greatest numbers of patients with chronic pain were seen in the general practice, with 50% (1991: 28%; p < 0.0001), and the fewest were seen in the surgical practice, with 32% (1991: 14%; p < 0.0001). In the oncology practice, 38.3% of all patients had chronic pain. Of all patients, 22.1% suffered from acute pain (1991: 25.3%). In both survey years, women suffered from chronic pain twice as often as men. [Table 1]. The open question about pain diagnosis could not be analysed because of missing answers. Further results of the individual specialists' offices will be presented in a separate paper.

### Characteristics of chronic pain

**Figure 1:**
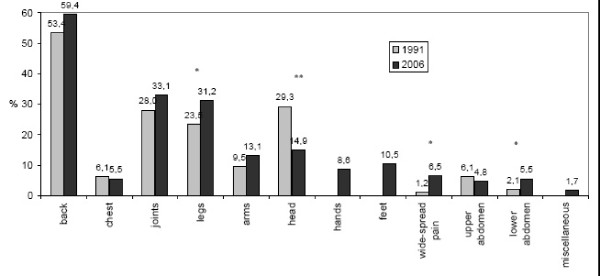
**Distribution of bodily locations of chronic pain in 1991 and 2006 (%)**. Multiple answers allowed, n1991 = 328, n2006 = 475 *p < 0.05, **p < 0.001.

The most frequent type of pain was musculoskeletal pain. The main locations of pain were back in 59.4% (1991: 53.4%) and joints in 33.1% (1991: 28.0%). Only 14.9% complained of headache (1991: 29.3%; p < 0.0001). [Fig. 1].

The number of patients suffering pain for more than five years was as high as 67.5% (1991: 51.5%; p < 0.0001), while 26% of patients recorded pain lasting longer than 20 years (1991: 16.7%; p = 0.0015).

A total of 73.2% of chronic pain sufferers estimated the intensity of their chronic pain to be between 6 and 10 on the numeric rating scale (NRS 0–10; 1991: 34.1%).

In both time periods, the factors most frequently activating chronic pain were physical strain in 72.6% (1991: 65.4%; p < 0.0001), weather conditions in 41% (1991: 49.4%; p = 0.0208) and psychological strain in 34.5% (1991: 36.9%).

The open question about the pain-causing diagnosis could not be analyzed due to missing answers.

**Figure 2:**
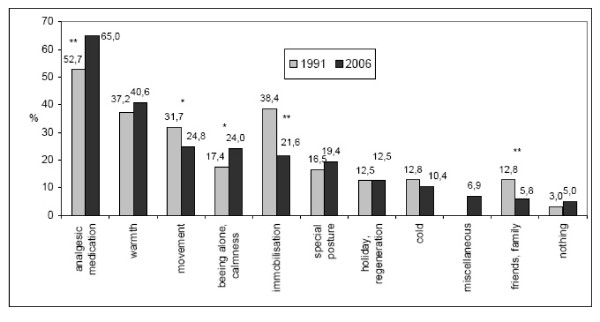
**Most helpful measures at relieving chronic pain (%)**. Multiple answers allowed, n1991 = 328, n2006 = 463 *p < 0.05; **p < 0.001.

Among own efforts at relieving pain, analgesic medication was considered the best method by 65% of the respondents (1991: 52.7%; p = 0.0005). [Fig. 2].

### Effects of chronic pain

**Figure 3:**
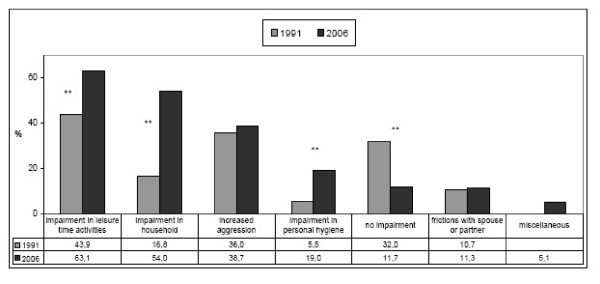
**Impairment of daily activities by chronic pain (%)**. Multiple answers allowed, n1991 = 328, n2006 = 452 *p < 0.05, **p < 0.001.

Impairment in everyday life caused by chronic pain was felt significantly more strongly than 15 years earlier. This became especially apparent during leisure in 63.1% (1991: 43.9%; p < 0.0001), housekeeping in 54% (1991: 16.8%; p < 0.0001) and personal hygiene activities in 19% (1991: 5.5%; p < 0.0001). In 14.3% of the patients, workdays were lost because of their chronic pain (1991: 19.2%). Only 11.7% did not feel any impairment from their chronic pain (1991: 32%; p < 0.0001). [Fig. 3].

Chronic pain had negative effects on the sleep of 66.3% of the patients (1991: 70.1%), and 24.8% were on sleeping pills because pain affected their sleep so much (1991: 34.8%).

### Previous therapies and their subjective effects

Only 6.3% of respondents with chronic pain had not yet had any specific pain treatment. The frequency of consulting a pain specialist had increased to 11.3% in 2006 (1991: 0.9%; p < 0.0001).

The methods used for pain therapy had increased: medication in 69.8% (1991: 51.2; p < 0.0001), injections in 51.2% (1991: 39.6%; p < 0.0001), acupuncture in 49.7% (1991: 5.8%; p < 0.0001) and operations in 21.2% (1991: 9.5%; p < 0.0001) were used against pain.

Medication was considered to be the most effective method in 34.4% (1991: 25.9%; p = 0.107), while 18.8% considered physiotherapy an efficient treatment (1991: 8.8%; p < 0.0001), 17.7% obtained pain relief from acupuncture (1991: 3.6%; p < 0.0001), and 24.7% of the patients did not consider any of these methods an efficient way of relieving pain (1991: 29.3%).

## Discussion

This study is the first to directly compare data on the changes in pain prevalence on randomly selected days in a comparable population in primary care, focussing on pain as the reason for outpatient health care visits. The investigation was designed as an independent, unselected random sample of outpatient health care users following the same model as in the first survey carried out in 1991 [9,12].

The point prevalence was unchanged from 15 years ago. Nearly every second patient consulted a primary care physician because of pain, and every fourth patient visited a doctor because of long-lasting pain. These figures illustrate the pain sufferer's high need for proper diagnosis and treatment.

In the last 15 years, the possibilities for pain treatment have definitely improved in Germany, both with regard to therapy options and the number of pain specialists. The period prevalence of chronic pain lasting longer than six months, however, has not decreased accordingly. Within these 15 years a significant improvement – a decrease in chronic pain – was expected. In these years the number of pain specialists and interdisciplinary pain clinics in Germany increased considerably [18,19]. The specialisation "special pain therapy" was introduced in 1996, bringing the first specialisation worldwide. However, these improvements, as well as the facilitation of pain therapy by the amendment of opioid regulations have obviously had no positive impact on the prevalence of chronic pain. Obviously, competence in diagnosis and treatment of pain has not changed.

The city of Bochum, with 380 000 inhabitants is an urban area in western Germany. All patients in Germany are primarily diagnosed and treated by their home physicians. It is up to the patient to decide which physician to use as a home physician. Patients can select any outpatient clinic for their home physician (general practitioner, neurologist, surgeon, internist), but the primary care consultation always takes place with one of these home physicians. Different practices were chosen during the investigations to include a wide spectrum of possible consultations for pain. Therefore our figures give a fairly exact picture of pain in primary care.

Our results are supported by previous and current studies. In collected data from Finland, the point prevalence of patients consulting a general practitioner due to pain was 40% [20]. Also a report from the USA indicated that more than one-third of adult appointments in a typical week in primary care involved patients with complaints of chronic pain [21].

There are no studies investigating trends of point prevalence of pain among health care users. The period prevalence of pain complaints was found to be stable in the adult Danish population over a five-year period [22]. However, the point prevalence of specific musculoskeletal pain symptoms over 40 years in the British population increased about 2- to 4-fold [23].

Among other possible reasons, this could be explained by an increase in the reporting or awareness of pain as the leading symptom. The number of pain patients is expected to rise. The demographic changes in industrial countries alone account for the increased average age of all patients in the study. Patients suffering from long-lasting pain are ten years older than those suffering from acute pain or those without pain. Age is one predisposing factor for chronic pain. Increasingly, "pain in the elderly" plays an important role in public discussions [24,25].

Limitations

To simplify the realisation of this survey, a one-dimensional definition of chronic pain by duration has been chosen. Certainly a multidimensional definition of pain would cover the pain problem better [26]. However, such a multidimensional approach is difficult in the mixed patient population and in the busy environment of primary care. Most importantly, in our study the main focus was pain as a symptom for which to seek primary care.

Data from open questions were excluded to guarantee comparability. Lack of answers led to insufficient data. It is assumed that the missing replies about profession were an issue of privacy and those about pain diagnosis were due to nescience.

We did not study the doctors' records. Medical consultation records instead of our surveys would give a false negative picture of the pain problem, because pain diagnoses very seldom occur. The International Classification of Diseases (ICD) still has no specific diagnosis for chronic pain. Pain occurs as angina pectoris, persistent somatoform disorder or trigeminal neuralgia [27]. Related to the ICD, pain is a minor health problem, because it is summarised under somatic disorders with the symptom of pain. This understanding contrasts with the modern view of pain as an illness on its own [28].

The inclusion of an oncology practice did not change the balance by increasing the pain prevalence. We have included these patients, because a criticism of our initial paper was the fact that cancer patients were omitted. It was suspected that this absence of cancer patients would have decreased the total prevalence. Surprisingly, fewer patients in oncology consulted the doctor due to pain. In the actual data from 2006 this assumption is not justified, as our figures demonstrate. We believe that comparability between the two studies was also provided by the inclusion of cancer patients.

The most prominent pain problem remains back pain, where simple diagnostic and therapeutic tools are neglected [4,29-31]. Pain in the musculoskeletal system is the main symptom for seeking a consultation with a primary care physician [32].

Our random sample has revealed that, in particular, patients who are unemployed, divorced or who left school after grade 9 have a greater tendency to suffer from chronic pain. Similar results have been published [33,34]. Such social parameters reflect the general development in modern societies. More people have reached higher education levels, and the divorce rate in Germany has increased from 30% to over 50% [35].

Pain had a considerable impact on the daily life of the patients concerned. The fact that 90% see themselves as impaired in their daily activities illustrates the extent of the restrictions due to pain. Emotional changes due to pain lead to high psychological strain and loss of quality of life [36]. In addition, there are severe consequences to be considered from a (socio-) economic point of view [37-39]. Nowadays, 90% of pain sufferers experience impairment of their jobs. Despite their pain, more people actually continued to go to work [40]. It can be anticipated that work efficiency is impaired by chronic pain, and this might be as important as the loss of working days [41]. Therefore, countrywide provision of multimodal pain therapy is needed [42], as well as effective early pain diagnosis for the prevention of chronic pain. The costs of both solutions have proven to be lower than the potential follow-up costs of a chronic pain disease or the costs of absenteeism and early retirement [2,39,43]. Only then it will be possible to take the pressure off the health care system in years to come.

These days pain causes more severe complaints and interferences. This assumption is supported by the fact that patients frequently complained of the psychosocial impacts of their chronic pain. Many patients with chronic pain continue working due to the increased pressure of the actual job market. The German health report presents fewer sickness leaves in the last decade [44]. Interestingly pain has not been mentioned as a symptom or as an illness in the German health report. Facing the fact that nearly every second patient makes use of primary care due to a pain problem underlines the need to make pain as an illness and a health problem more visible. Moreover, there is possibly an indeterminate number of patients who do not consult a doctor because of fear of unemployment.

The average duration of pain in chronic pain sufferers was 10.7 years [17]. Patients fail to receive adequate diagnosis and treatment of pain in primary care, before they attend a specialist after too many years of mismanagement [45]. Consequently, psychological therapies, medication and alternative techniques are now used more often. This increased need for different treatment approaches and the rising number of patients suffering from intense pain could be explained by a higher degree of chronic suffering over the last decade. Longitudinal studies confirm this observation [46,47]. However, the increased use of different approaches does not result in improved pain relief, as the respondents considered single measures rather ineffective. Every fourth pain patient is displeased with the effect of therapy, which is a slight improvement compared to our previous results [9,12]. The rate of pain treatment success is low, and confounding factors – psychological and social – contribute to a purely somatic picture of pain [7]. Awareness of non-somatic factors is important to avoid chronification.

However, only a few patients have received pain treatment properly adjusted to their needs. Less than 24.7% of patients consider any of their therapies satisfactory in reducing their chronic pain [48]. Surprisingly, neither a pain clinic nor pain specialists were able to fully meet their needs.

In our study, only a few patients who visited the practices were unwilling to take part and were excluded. The personal contact in the practice waiting rooms may have been of help, as the rate of refusal was far lower than in other pain prevalence investigations using telephone interviews or postal questionnaires, where the rates of refusal were 29.2% and 64.1%, respectively [49,50].

Pain plays a central role in the health care system, and is one of the most frequent reasons for consulting a doctor. There is a strong association between pain and the greater use of health care services [51,52]. Primary care is the most frequent care provider for pain [53]. However, most health care systems are not sufficiently prepared to meet these demands. If this fundamental problem is not solved, the process of chronification of pain proceeds. To distinguish pain as an illness on its own, a careful diagnosis is essential [28,54].

A solution to the continuing problem of chronic pain is only a careful undergraduate and postgraduate training in pain diagnosis and treatment [6,55]. Pain as a major health problem – possibly *the *major health problem – is still not obligatory in undergraduate training in Germany.

## Conclusion

Pain is the reason for primary health care utilisation in nearly half of all cases. The family doctor plays a key role, being the first contact person for the patient [3], and the most important one for preventing the chronification of pain. Also, for family physicians, a multidimensional approach is required to address the problem in the community [56]. The treatment of pain usually comes too late. Prevention of chronic pain is the central issue, and improvement can only be made by improving quality at the primary care level. Our figures demonstrate that there is still a huge deficit in this area of care.

## Competing interests

The authors declare that they have no competing interests.

## Authors' contributions

CHF was the principal investigator, attended the questionnaire survey, did the statistical analyses, and drafted the manuscript. AWS contributed to the statistical analyses and participated in the drafting of the manuscript. MWZ was responsible for the concept, made a critical revision of the manuscript. All authors have read and approved the final manuscript.

## Pre-publication history

The pre-publication history for this paper can be accessed here:

http://www.biomedcentral.com/1471-2458/9/299/prepub

## Supplementary Material

Additional file 1 **German Questionnaire**. The file provides the German questionnaire used in this study. Click here for file
