# Supplementary material for: Chronic pain in primary care. German figures from 1991 and 2006
Source: BMC Public Health. 2009 Aug 18;9:299. doi: 10.1186/1471-2458-9-299 (PMC2744700; doi:10.1186/1471-2458-9-299)
Supplement: Additional file 1 — German Questionnaire. The file provides the German questionnaire used in this study. [file 1471-2458-9-299-S1.pdf]

Christine Frießem
Datum ☐☐ ☐☐ ☐☐ Praxisnummer ☐ ☐☐☐☐

Fragebogen

Teil A:

1. *Geschlecht:* ☐ weiblich ☐ männlich

2. *Alter:* ________Jahre

3. *Sind Sie:* ☐ ledig ☐ verheiratet ☐ verwitwet ☐ geschieden?

4. *Haben Sie Kinder?* ☐ nein ☐ ja Wie viele?________________

5. *Wohnen Sie alleine?* ☐ nein ☐ ja

6. *Welche Schulausbildung haben Sie?* ☐ Hauptschule ☐ Realschule ☐ Abitur ☐ Sonstiges

7. *Welche Tätigkeit/ Beruf üben Sie aus?* ____

☐ berufstätig ☐ derzeit ohne Beschäftigung

☐ Rentner ☐ Hausfrau/ -mann

8.a *Sind Schmerzen der Grund des heutigen Arztbesuches?* ☐ ja ☐ nein

8.b *Unter welchen Schmerzen leiden Sie?* ☐ keinen

☐ akuten (seit Stunden oder Tagen auftretender Schmerz)

☐ chronischen (wiederkehrender oder anhaltender Schmerz länger als 6 Monate)

Wenn Sie an chronischen Schmerzen leiden, füllen Sie bitte auch Teil B aus.

Herzlichen Dank!

Teil B:

9. *Wie lange leiden Sie bereits an chronischen Schmerzen?*

☐ 6 bis 12 Monate ☐ 1-2 Jahre ☐ 2-5 Jahre

☐ 5-10 Jahre ☐ 10-20 Jahre ☐ mehr als 20 Jahre

10. *Wo haben Sie Schmerzen? (*Mehrfachauswahl möglich.)

☐ Rücken ☐ Brust ☐ Gelenke

☐ Beine ☐ Arme ☐ Kopf

☐ Hand ☐ Fuß ☐ Ganzer Körper

☐ Oberbauch ☐ Unterbauch

*Nähere Beschreibung:*____

11. *Wie oft haben Sie Schmerzen?*

☐ dauernd ☐ mehrmals täglich

☐ alle paar Tage ☐ alle paar Wochen

12. *Wie stark sind Ihre Schmerzen?*

Bleiben Ihre Schmerzen konstant, so markieren Sie nur eine Zahl in der Tabelle von 0 bis 10. Bei Schmerzen schwankender Intensität zeichnen Sie bitte ein Minimum und ein Maximum ein.

(0= keine Schmerzen, 10= stärkste Schmerzen)

0	1	2	3	4	5	6	7	8	9	10

13. *Wie lange dauern die Schmerzen an?* (Mehrfachauswahl möglich.)

☐ minutenlang ☐ stundenlang

☐ tagelang ☐ ständig vorhanden

14. *Haben sich die Schmerzen im Laufe der Zeit verändert?* (Mehrfachauswahl möglich.)

☐ nein ☐ stärker geworden ☐ seltener geworden

☐ schwächer geworden ☐ häufiger geworden

15. *Was sehen Sie als Ursache für ihre Schmerzen?* (Mehrfachauswahl möglich.)

☐ Verschleiß ☐ seelische Belastung ☐ Unfall

☐ Beruf ☐ Operation: _________________ ☐ Kriegsverletzung

☐ Vererbung ☐ Krankheit: _________________

☐ Sonstiges:_____________________

16. *Wann treten die Schmerzen auf?* (Mehrfachauswahl möglich.)

☐ mit Auslöser, Einwirkung ☐ ohne Auslöser, spontan

17. *Was kann Ihre Schmerzen auslösen oder verstärken?* (Mehrfachauswahl möglich.)

☐ körperliche Belastung ☐ psychische Belastung, Stress ☐ Wetter
☐ Stimulation (Alkohol, Zigaretten, Kaffee) ☐ Entspannung
☐ Tageszeiten: ☐ Morgens ☐ Mittags ☐ Abends ☐ Nachts
☐ Sonstiges:__________________

18. *Was kann Ihre Schmerzen lindern?* (Mehrfachauswahl möglich.)

☐ Medikamente ☐ Wärme ☐ Ruhigstellung
☐ alleine sein, Ruhe ☐ Kälte ☐ Bewegung, Sport
☐ Freunde, Familie ☐ besondere Körperhaltung ☐ Urlaub, Erholung
☐ gar nichts ☐ Sonstiges: _________________

19. *Wie wirken sich die Schmerzen auf Ihren Alltag aus?* (Mehrfachauswahl möglich.)

☐ keine Beeinträchtigung ☐ vermehrt Aggression, Ungeduld, Nervosität
☐ Beeinträchtigung im Haushalt ☐ Beeinträchtigung bei Körperpflege
☐ öfter Spannungen mit Mitmenschen ☐ eingeschränkte Freizeitgestaltung
☐ Sonstiges: ____________________________

20.a *Schlafen Sie aufgrund der Schmerzen schlechter?* ☐ ja ☐ nein

20.b *Wenn ja, nehmen Sie Medikamente zum Schlafen?* ☐ ja ☐ nein

20.c *Wenn ja, welche?* ____

21. *Wenn Sie berufstätig sind: beeinträchtigen die Schmerzen Ihren Beruf?*
(Mehrfachauswahl möglich.)

☐ tageweise Fehlen bei der Arbeit ☐ Rentenantrag gestellt
☐ Berentung aufgrund der Schmerzen ☐ Berufswechsel
☐ Teilzeitarbeit ☐ Konzentrationsschwäche
☐ keine berufliche Beeinträchtigung

22. *Wie wirken sich die Schmerzen auf Ihre Lebensqualität aus?*
(0= keine Beeinflussung, 10= starke Beeinflussung)

0	1	2	3	4	5	6	7	8	9	10

23. *Welche Fachärzte haben Sie wegen Ihrer Schmerzen aufgesucht?*
(z.B. Allgemeinmedizin, Chirurgie, Neurologie, Orthopädie, Psychologie, Heilpraktiker, Schmerzklinik etc.)

____

24. *Kennen Sie die Diagnose Ihrer Schmerzen?*

☐ nein ☐ ja, meine Diagnose lautet: ____________________________

25. *Welche Therapien haben Sie aufgrund der Schmerzen gemacht?* (Mehrfachauswahl möglich.)

☐ keine

☐ psychologische Therapie ☐ Kur ☐ Operation

☐ Medikamente ☐ Spritzen ☐ Akupunktur

☐ Krankengymnastik ☐ physikalische Therapie (z.B. Thermotherapie)

☐ Massage ☐ Sonstiges: ____________________________

26. *Welche Behandlung hat Ihre Schmerzen am wirkungsvollsten gelindert?* (Mehrfachauswahl möglich.)

☐ keine

☐ psychologische Therapie ☐ Kur ☐ Operation

☐ Medikamente ☐ Spritzen ☐ Akupunktur

☐ Krankengymnastik ☐ physikalische Therapie (z.B. Thermotherapie)

☐ Massage ☐ Sonstiges: ____________________________

27.a *Welche Medikamente nehmen Sie derzeit gegen Ihre Schmerzen ein?*

____

____

27.b *Nach welchem Schema nehmen Sie diese Medikamente ein?* (Mehrfachauswahl möglich.)

☐ nach Bedarf: welche? ____

☐ nach festem Zeitplan: welche? ____

28. *Haben Sie zuvor andere Medikamente gegen Schmerzen verschrieben bekommen? Wenn ja, welche?*

____

____

29. *Welche Nebenwirkungen haben Sie bei der Einnahme von Schmerzmedikamenten bisher bemerkt?* (Mehrfachauswahl möglich.)

☐ keine ☐ Verstopfung ☐ Schläfrigkeit

☐ Magenbeschwerden ☐ Allergie ☐ Kopfschmerzen

☐ Abgeschlagenheit ☐ Abhängigkeit ☐ Sonstige:__________

30. *Haben Sie bereits im Jahre 1990/ 1991 an einer ähnlichen Befragung teilgenommen?*

☐ ja ☐ nein

<u>Herzlichen Dank für ihre Mitarbeit!</u>
